# Piracy and Obstinacy

**Published:** 1886-02

**Authors:** 


PIRACY AND OBSTINACY.—DANIEL'S TEXAS MEDICAL JOURNAL accuses the *American Medical Journal* of the unpardonable sin of plagiarism. This is a grave charge, and, unless refuted, will seriously affect the exchange list of the accused.—*St. Joseph Medical Herald.*

The *American Medical Journal*, under head of "Original Communications," produced two of the papers which had been written for, and published in this journal; and, in addition to the mention of it, alluded to by our St. Joseph contemporary, we took occasion to write to Dr. Pitzer, the editor, calling his attention to the matter. To our letter the gentleman did not even accord us the courtesy of a reply. Exchanges can govern themselves by these facts.

